# Advances on Non-Genetic Cell Membrane Engineering for Biomedical Applications

**DOI:** 10.3390/polym11122017

**Published:** 2019-12-05

**Authors:** Lisha Liu, Hongliang He, Jianping Liu

**Affiliations:** 1 Department of Pharmaceutical Sciences, College of Pharmacy, University of Michigan, 428 Church St, Ann Arbor, MI 48109, USA; lishaliu@med.umich.edu; 2 Department of Pharmaceutics, China Pharmaceutical University, Nanjing, Jiangsu 210009, China

**Keywords:** cell-based therapeutics, whole-cell-based therapeutics, cell membrane-derived therapeutics, extracellular vesicles, non-genetic cell surface engineering, targeted drug delivery, nanoparticles

## Abstract

Cell-based therapeutics are very promising modalities to address many unmet medical needs, including genetic engineering, drug delivery, and regenerative medicine as well as bioimaging. To enhance the function and improve the efficacy of cell-based therapeutics, a variety of cell surface engineering strategies (genetic engineering and non-genetic engineering) are developed to modify the surface of cells or cell-based therapeutics with some therapeutic molecules, artificial receptors, and multifunctional nanomaterials. In comparison to complicated procedures and potential toxicities associated with genetic engineering, non-genetic engineering strategies have emerged as a powerful and compatible complement to traditional genetic engineering strategies for enhancing the function of cells or cell-based therapeutics. In this review, we will first briefly summarize key non-genetic methodologies including covalent chemical conjugation (surface reactive groups–direct conjugation, and enzymatically mediated and metabolically mediated indirect conjugation) and noncovalent physical bioconjugation (biotinylation, electrostatic interaction, and lipid membrane fusion as well as hydrophobic insertion), which have been developed to engineer the surface of cell-based therapeutics with various materials. Next, we will comprehensively highlight the latest advances in non-genetic cell membrane engineering surrounding different cells or cell-based therapeutics, including whole-cell-based therapeutics, cell membrane-derived therapeutics, and extracellular vesicles. Advances will be focused specifically on cells that are the most popular types in this field, including erythrocytes, platelets, cancer cells, leukocytes, stem cells, and bacteria. Finally, we will end with the challenges, future trends, and our perspectives of this relatively new and fast-developing research field.

## 1. Introduction

Cell therapy is the transfer of intact and live cells from the patient (autologous cells) or a donor (allogeneic cells) into a patient to replace or repair damaged tissue and/or cells. The early record of cell therapy could data back to 1931, when Swiss doctor Paul Niehans attempted to cure a patient by injecting material from calf embryos. With the evolving research and emerging innovative technologies, various cell types have been engineering as novel therapeutics for a variety of diseases and conditions [1,2,3]. Some of the cells that may be used include erythrocytes, platelets, cancer cells, leukocytes, stem cells, and bacteria. Among these, erythrocytes exhibit high biocompatibility when used as autologous cells and as therapeutic carriers [4,5]. Based on their vital roles in hemostasis and thrombosis, and their recent identified importance in the development and metastasis of tumors, platelets have been extensively studied as novel drug delivery carriers for enhanced efficacy [6,7]. The whole cancer cells as native antigens have been demonstrated to boost the organism’s immune system to fight against tumor [8,9]. Leukocytes have recently received many achievements in the treatment of various diseases, particularly in cancer, which explore the native migration of leukocytes subpopulations (such as monocytes/macrophages) to inflamed tissues or the innate ability of leukocyte subpopulations (such as lymphocytes) to elicit antitumor immunity [10,11]. Hematopoietic stem cells (HSC) transplantation (also called bone marrow transplant) is the most frequently used cell therapy to treat a variety of blood cancers and hematologic disorders [12]. Mesenchymal stem cells (MSC) have emerged as a cornerstone of regenerative medicine, due to their diverse spectrum of differentiation into cardiomyocytes, cartilage, or neurons, and the ease of culturing quantities appropriate for clinical application [13]. In addition, MSC can suppress lymphocyte proliferation, which have been utilized clinically to treat lymphocyte cell-dependent diseases, such as amylotrophic lateral sclerosis, acute graft-versus-host disease, or transplant rejection [12,14,15]. As the nature of pathogens is to penetrate through the tissue to invade a host and stay alive, some bacteria (such as salmonella) were found out to move between cells to reach tumors, which has been engineered as therapeutics or drug delivery systems for tumor treatment [16,17].

With the advance and interaction of the multiple disciplines, such as engineering, materials, biochemical, and physical science, cell-based therapeutics have evolved from the whole-cell-based therapeutics to some novel therapeutic modalities, including cell membrane-derived therapeutics and extracellular vesicles [10,18,19]. Cell membrane-derived therapeutics, such as cell membrane-derived vesicles and cell membrane-camouflaged nanoparticles, are directly leveraging cell membranes as a means of bestowing nanoparticles with native cells’ distinctive biological capabilities [8,20]. Extracellular vesicles, such as exosomes, undertake various pathophysiological roles such as shuttling proteins and genetic information in cell-to-cell communications and stimulating adaptive immune responses. Extracellular vesicles are recently employed as a promising class of nanoparticles for a broad range of biomedical applications [21,22]. Both of them are constructed by substances derived or collected from cells; however, they are more robust to most mankind engineering processes and chemical modifications, compared to the relatively fragile structure of whole cells [23].

Despite the impressive advances described above, cell-based therapeutics still have multiple hurdles to overcome before they can be widely adopted for clinical use with a better efficacy [10,24]. One of the most important challenges is the inability to control the transport fate of injected cells in vivo, particularly for increasing the percentage of cells reaching target tissues and decreasing the off-target accumulation. Therapeutic cells transplanted into a recipient’s body may encounter undesired host immune responses and often lose their therapeutic activity due to the protective immune surveillance [25]. Another key issue is that loading drug or drug-loaded nanoparticles into cells may cause damage to the cell membrane and alter the cell phenotype, even being cytotoxic to carrier cells themselves under some cases [26].

In an attempt to circumvent the above limitations associated with cell-based therapeutics, cell membrane engineering is considered to be one of the most promising solutions to improve the therapeutic feasibility and effectiveness of cells [26,27]. Cell membrane engineering might include modifying the cells with some active molecules for therapeutic purposes, drug-loaded nanoparticles for payload depot, targeting the elements that are capable of enhancing targeted delivery, and signaling molecules for modulating immune reaction.

Cell membrane engineering has been generally achieved by two major methods: genetic engineering and non-genetic engineering [28,29]. Genetic engineering is the genetic incorporation of exogenous genetic material into the cell, where it encodes an artificial cell surface receptor that targets an antigen of interest. Genetic engineering has brought up two recent the Food and Drug Administration (FDA)-approved cell-based therapeutics, Kymriah and Yescarta [30]. Although genetic engineering is a robust modification technique, it has a number of significant drawbacks. One important issue is associated with the gene delivery vector. Viral vectors possessing high gene transfection efficiency have a high risk of genetic integration that may trigger immunogenic response and lead to tumorigenesis. In contrast, non-viral gene carriers show a better safety property; however, they always suffer rather low in vivo transfection efficiency compared to viral vectors. The other issues also have to be considered, including the time-consuming process of genetic engineering and that not all cell types are amenable to genetic modification. Due to the huge differences from non-genetic engineering strategies (mainly discussed in this review), genetic engineering will not be included in the scope of this review.

**Figure 1:**
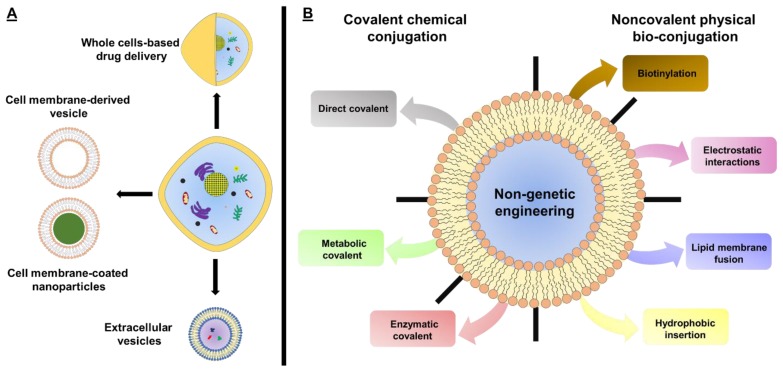
(**A**) Depiction of some cell-based therapeutic strategies, including whole-cell based drug delivery, cell membrane-derived vesicles or/-coated nanoparticles, and extracellular vesicles; (**B**) Overview of approaches currently used in non-genetic cell surface engineering, mainly consisted of covalent chemical conjugation and noncovalent physical bio-conjugation.

Alternative to genetic engineering, non-genetic cell surface engineering offer more transient and reversible modifications to control cellular functions. Non-genetic cell surface engineering can be broadly divided into covalent chemical conjugation and noncovalent physical bioconjugation. Those different non-genetic cell surface engineering strategies are summarized in many recent reviews [10,19,25,26,31,32,33,34,35]. Among these, the non-genetic cell surface engineering that is used to modulate and investigate cell functions and control cell–cell and cell–microenvironment interactions can be found in previous review articles [29,32,34]. The application of non-genetic cell surface engineering to improve the therapeutic potential of whole-cell-based therapeutics has been reviewed previously [10,19,26,31]. The non-genetic engineering of extracellular vesicles has been discussed in recent reviews [21,22]. Nevertheless, to our best knowledge, most of the review articles in this field only focus on the applications of these strategies for whole-cell-based therapeutics (live cells) or extracellular vesicles. None of them discussed the advancement on the application of non-genetic surface engineering in some novel cell-based therapeutics (nonliving cells, such as cell membrane-derived nanovesicles and cell membrane-coated nanoparticles). Hence, the comprehensive overview and latest update of application of those technologies in diverse cells or cell-based therapeutics are still scarce. The novelty of this review attempts to highlight the latest progresses of those non-genetic engineering strategies in various cells and cell-based therapeutics, including whole-cell-based therapeutics, cell membrane-derived nanovesicles, and cell membrane-coated nanoparticles as well as extracellular vesicles. The first section of this review will briefly summarize each type of non-genetic cell surface engineering technique. Specifically, covalent chemical conjugation can be further categorized into direct conjugation (amine reactive strategies and thiol reactive strategies) and indirect conjugation (enzyme-mediated strategies and metabolic-mediated strategies); noncovalent physical bioconjugation could be grouped into biotinylation, electrostatic interaction, and lipid membrane fusion as well as hydrophobic insertion. In the following sections, surface engineering on different cells or cell-based therapeutics in various biomedical applications will be largely exemplified, which include whole-cell-based therapeutics, cell membrane-derived therapeutics (cell membrane-derived nanovesicles and cell membrane-coated nanoparticles) and extracellular vesicles (main contexts schematically shown in Figure 1). To the end, the challenges and opportunities in this field will be discussed.

## 2. Non-Genetic Surface Engineering Strategies

### 2.1. Covalent Chemical Conjugations

Direct covalent modification is the most straightforward method that takes advantage of surface-exposed functional groups focusing on the primary amine groups (–NH_2_) and thiol groups (–SH) that are present in cell surface proteins [25,28]. *N*-hydroxysuccinimide (NHS) ester is widely used for the modification of primary amines on cell membranes. NHS-ester-activated compounds react with primary amines in slightly alkaline conditions (pH 7.2–8.5) for 0.5–4 h at room temperature or 4 °C to form amide bonds with the release of NHS that can be removed easily by dialysis or desalting. Most common covalent reactions for cell surface-exposed active thiol groups (–SH) involve maleimide-activated molecules, which specifically react with thiol groups at near neutral conditions (pH 6.5–7.5) to form stable thioether linkages.

Indirect covalent modifications mostly include metabolic-mediated modifications and enzymatic-mediated modifications. For metabolic-mediated modifications, a chemically reactive moiety is first incorporated into target biomolecules that are known as the “chemical reporters” by the endogenous machinery of the cell. In the second step, the reporter group is covalently reacted with a target functional biomolecules through bio-orthogonal ligation (or click chemistry) [33,36]. The most common chemical reporters, including azides and ketones, are used to metabolically incorporate to the cell surface by unnatural sialic acid biosynthesis. The corresponding biorthogonal ligations of azide are aryl phosphine, alkyne, and cycloalkyne, while the reaction of ketone requires hydrazide. The enzymatic-mediated modifications are that these reactive groups must be generated or exposed through enzymatic treatment of the originally existing cell surface molecules. For example, through being enzymatically mediated by galactose oxidase, the limited presence of carbonyl groups on cell surfaces are enhanced to enough surface concentration for efficient covalent chemical modifications, which are mediated via covalently reacting with hydrazide to form hydrazone bonds or reacting with alkoxy amine to form oxime bonds.

### 2.2. Noncovalent Physical Bioconjugations

Biotinylation is a process involving biotin that has been first conjugated or modified on the cell membrane and can be subsequently functionalized through avidin (or avidin analogs streptavidin and neutravidin). The high affinity biotin–avidin interaction (*K*_d_ = 1 × 10^−15^ M) is highly resistant to some harsh denaturing conditions, including heat, pH, and organic solvents. Hence, biotinylation has promising applications in cell surface engineering [35].

Electrostatic interaction utilizes the opposite charge interaction between the negatively charged cell membrane and the positively charged modifying materials. The negative charges of the cell outer surface result from the presence of sialic acid residues on glycoproteins or negatively charged proteins anchored to the cell membrane. One popular application of electrostatic interaction is the polyelectrolyte modified cell assembly, which is beneficial for tissue regeneration engineering. Specifically, cationic modifying materials first bind onto the negatively charged cell surface; then, the cell surface will turn to positive charge. After a certain number of cycles (layer-by-layer), multilayered structure assemblies with tunable robustness may be generated, creating a defined microenvironment for cell differentiation and proliferation [37].

Lipid membrane fusion is mediated by a spontaneous lipid leaflet fusion process, which generally occurs between two distinct lipid bilayer-directed structures (such as the liposome and cell membrane). When a functional group-containing liposome is applied to the cell membrane, those functional groups will be presented onto the cell surface through the membrane fusion process. This method also has some critical roles in many important cellular processes (such as phagocytosis and exocytosis) and has been intensely investigated for cell surface engineering [26].

Hydrophobic insertion is the complementary interaction between the hydrophobic residues in modifying substances (such as lipids and proteins) and the hydrophobic zones in cell membrane. When modifying substances conjugated with an appropriate hydrophobic anchor are admixed with cultured cells, the hydrophobic moiety can spontaneously insert into the hydrophobic zone between the lipid bilayer, thus anchoring the conjugated cargoes on the cell surface [31].

## 3. Application of Cell Surface Engineering in Different Cells or Cell-Based Therapeutics

### 3.1. Erythrocytes

Erythrocytes also called red blood cells (RBC) are the most abundant type of blood cells in the body. Some crucial self-markers on the surface allow erythrocytes to remain in circulation for a long period of time (~120 days lifespan in humans) [4,5,38]. Moreover, aged or damaged RBC are completely biodegradable in reticuloendothelial system (RES) without the generation of toxic by-products [39]. Those above unique merits make RBC potentially biocompatible as delivery carriers for a number of bioactive substances, such as anti-inflammatory [40,41], antiviral [42], anti-neurodegenerative [43], and anti-cancer drugs [44].

**Figure 2:**
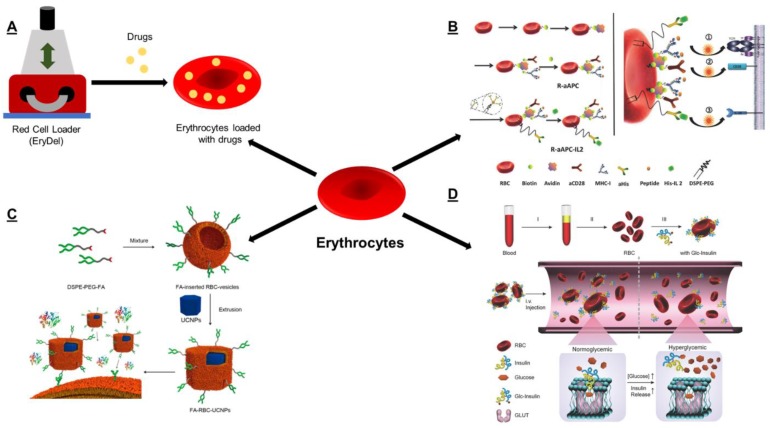
(**A**) Schematic illustration to show the process of Red Cell Loader to load drugs into erythrocytes. (**B**) Schematic presentation to show the fabrication of red blood cell (RBC)-based artificial antigen-presenting cells (APC) modified with pMHC-I, aCD28, and IL2, as well as the mechanism of activating CD8^+^ T cells, reprinted with permission from [51]. (**C**) The modification of the RBC membrane with some tumor targeting ligands (DSPE-PEG-FA) enhanced tumor accumulation, when coated onto the surface of upconversion nanoparticles (UCNPs) to generate FA-RBC-UCNPs. Adapted and reprinted with permission from [52]. (**D**) Schematic depiction of engineering RBC membrane with glucose derivative-conjugated insulin for smart blood glucose control. Reprinted with permission from [53]. DSPE: phosphorylethanolamine, FA: folate, PEG: polyethylene glycol.

Due to the absence of a nucleus and some subcellular organelles, the encapsulation of those biological and chemical agents into RBC has been highly explored with great achievements. The biotechnology company EryDel SpA’s proprietary platform technology (Figure 2A), Red Cell Loader (RCL), is an easy to use, non-invasive and automatic bedside medical device to encapsulate small and large therapeutic molecules such as therapeutic enzymes or recombinant proteins into patients’ erythrocytes. Those drug-loaded erythrocytes are reinfused into patients providing better tolerability, reduced immunogenicity, and prolonged half-life in circulation as well as predictable vascular distribution. EryDel SpA has a rich pipeline of preclinical programs that use RCL technology for the treatment of some rare diseases. They include erythrocyte loaded with dexamethasone sodium phosphate (EryDex) for the treatment of ataxia telangiectasia [45], erythrocyte-encapsulated thymidine phosphorylase (EE-TP) for the treatment of mitochondrial neurogastrointestinal encephalomyopathy [46], and erythrocyte-encapsulated phenylalanine hydroxylase (Ery-PAL) for the genetic disorder phenylketonuria [47]. Among, EE-TP is currently under phase 2 clinical study (NCT03866954). A pivotal phase 3 study (ATTeST) is currently ongoing with EryDex (NCT02770807).

Although the internal loading of drugs within RBC is now entering clinical use, there are some potential problems associated with the internal drug-loading procedure. For example, those drug-loading procedures might compromise cell membrane integrity, thus leading to insufficient circulatory survival; in addition, cellular drug encapsulation usually shows an incomplete or uncontrolled drug release. As a complementary approach for the production of RBC drug carriers, cell surface engineering has been investigated for efficient drug loading or better efficacy based on the large surface-to-volume ratio of and abundant surface functional groups on RBC (amino acids, thiol groups, sugars, and lipids) [42,48]. Some biological molecules can be linked to the exterior of the RBC through direct covalent chemical conjugation [39,49,50]. Initial studies of membrane-engineered RBC aimed to reduce the immunogenicity of transfused allogeneic erythrocytes. Polyethylene glycol (PEG) or hyperbranched polyglycerol were chemically or physically coupled on the membranes of erythrocytes. This camouflage modification significantly extended their circulation time and did not change the morphological and functional properties of erythrocytes, which are beneficial for blood transfusion in clinic [19].

In addition to RBC transfusion in the treatment of anemic patients, the RBC membrane engineering has been widely applying to drug delivery. In order to overcome these limitations associated with tissue-type plasminogen activators (tPA), including rapid clearance (incomplete effectiveness), bleeding, and extravascular toxicity, Dr. Muzykantov’s lab conjugated tPA to isolated RBC to prolong tPA’s lifespan in circulation and achieve excellent fibrinolytic activity. To achieve this, they coupled tPA to the surface of RBC (RBC-tPA) using streptavidin–biotin as a cross-linker [54]. The amount of RBC-bound tPA that remained in the bloodstream after bolus injection of RBC-tPA was markedly greater than that remaining after bolus injection of free tPA. To be specific, less than 10% of injected free tPA was detected 1 h after injection, while 80%–85% of the injected dose of RBC-tPA remained in circulation 2 h after injection. Moreover, in a mouse model of pulmonary emboli, the fibrinolytic activity of RBC-tPA in lysing nascent over preexisting pulmonary emboli was at least 10-fold longer than that of free tPA. Those RBC manipulations, including RBC isolation, *ex vivo* drug loading, and the reinfusion of those drug-coupling RBCs are applicable to some disease settings where the risk of thrombosis can be expected and transfusion is part of routine clinical care. However, those complicated RBC manipulations may be impractical in most settings where the diseases are urgently needed for intervention, such as thromboprophylaxis characterized by a high imminent risk of thrombosis. In order to improve the speed, safety, and utility of this RBC-based drug delivery strategy, Dr. Muzykantov’s lab conjugated tPA to a monoclonal antibody (mAb) against complement receptor type 1 (CR1) that was expressed primarily on human RBC using a chemistry-based bifunctional cross-linking pair, including succinimidyl 4-(N-maleimidomethyl) cyclohexane-1-carboxylate (SMCC) and N-succinimidyl S-acetylthioacetate (SATA) [55]. After being administered into blood, the anti-CR1-directed tPA rapidly bound to RBC in blood through the antibody–receptor interaction. The animal studies demonstrated that this antibody-directed tPA loading did not compromise RBC’s biocompatibility and survival in vivo, which showed identical kinetics of blood clearance and organ distribution to the native RBC. In addition, RBC-tPA markedly prolonged tPA’s half-time in circulation, accelerated the lysis of venous thrombi, and prevented the formation of stable occlusive carotid arterial thrombi, suggesting a safe and effective means of fibrinolytics delivery.

In addition to a wide application of RBC as long-circulating drug carriers, RBC have also been subjected to surface engineering for other applications. Dr. Liu and his group first loaded the chemotherapeutic drug doxorubicin into RBC via the hypotonic dialysis method [56]. Later, they attached iron oxide nanoparticles precoated with a photosensitizer (chlorine e6) to doxorubicin-loaded RBC to obtain a biocompatible multifunctional platform, which enabled imaging-guided combined photodynamic and chemotherapy, thus achieving excellent synergistic antitumor effects without appreciable toxic side effects. With the advancement of cancer immunotherapy, his group proceeded to successfully engineer the surface of RBC to generate artificial antigen-presenting cells (APC) to stimulate T cells and induce antitumor immune responses [51]. In their work, sulfo-NHS-biotin was first reacted with the prime amine groups on the surface of RBC to obtain biotinylated RBC. Biotinylated RBC were further reacted with streptavidin to generate the streptavidin modifying RBC. Some biotinylated active biologics, including antigen peptide (OVA257−264, SIINFEKL)-loaded major histocompatibility complex-I (pMHC-I) and an anti-CD28 antibody, were engineered onto the surface of RBC to obtain artificial APC by a biotin–avidin bridge (Figure 2B). The surface engineering did not show any negative effect on the RBC integrity. Those RBC-based artificial APC showed favorable antigen-specific CD8^−^ T cell activation functions, enhanced T cell proliferation, and increased inflammatory cytokines secretion (interferon gamma, IFN-γ and tumor necrosis factor alpha, TNF-α). Nevertheless, those results were only obtained from *in vitro* cell studies; the antitumor efficacy of this engineered RBC-based APC in animals is yet to be tested.

Inspired by those remarkable properties, the RBC membrane has also been intensively engineered by researchers to prepare a nanovesicle or to camouflage nanomaterials (such as nanoparticles) to mimic the chemical characteristics and biological functions of RBC. In order to improve the cancer-targeting property of RBC membrane-directed drug delivery systems, Dr. Zhang and his group modified the RBC membrane with some tumor targeting ligands [57]. His group exploited two tumor targeting ligand–linker–lipid conjugates (folate–PEG–lipid and AS1411–PEG–lipid) and then inserted them into RBC membrane ghosts, respectively. The resulting ligand-functionalized RBC membranes were employed to coat a polymeric nanoparticle core to form functional RBC membrane-cloaked nanoparticles, which showed remarkable receptor-specific targeting against model cancer cell lines. Guo et al. isolated the RBC membrane and inserted DSPE–PEG–mannose into those isolated RBC membrane via the “hydrophobic insertion” method [58]. Mannose-inserted RBC membrane further coated poly(lactic-co-glycolic acid) (PLGA) nanoparticles, which encapsulated an antigenic peptide and a Toll-like receptor 4 agonist. Those engineered RBC membrane-coated nanovaccines actively targeted APC (especially macrophages and dendritic cells) in lymphatic organs via mannose receptor-mediated cellular uptake. The results showed that this novel nanovaccine showed higher APC uptake and retained in the lymph nodes for a longer time. Furthermore, due to enhanced CD8^+^ T cell response and IFN-γ secretion, this nanovaccine retarded the tumor-occurring time, suppressed the tumor growth, and inhibited the tumor metastasis in all prophylactic, therapeutic, and metastatic melanoma models. Similarly, Rao et al. modified the RBC membrane with cancer targeting ligand folate (FA) via inserting DSPE-PEG-FA into RBC membrane, which further coated onto the surface of upconversion nanoparticles (UCNPs) to generate FA-RBC-UCNPs that showed enhanced tumor imaging and good biocompatibility in vivo (Figure 2C) [52].

In addition to cancer therapy, Dr. Gu and his team used the surface engineering of RBC to design a smart glucose-responsive insulin delivery system, mimicking the function of pancreatic β-cells to achieve the precise control of blood glucose [53]. In their delivery system (Figure 2D), a glucose derivative glucosamine was conjugated to insulin, which efficiently bound to glucose transporters on RBC membranes. Due to the competitive interaction of free glucose with glucose transporters, insulin-coupled RBC rapidly released insulin from RBC mediated by the displacement of free glucose under the high glucose level (or hyperglycemia). Moreover, this smart insulin delivery was simplified and validated by directly utilizing the above insulin-coupled RBC membrane to coat PLGA nanoparticles.

### 3.2. Platelets

**Figure 3:**
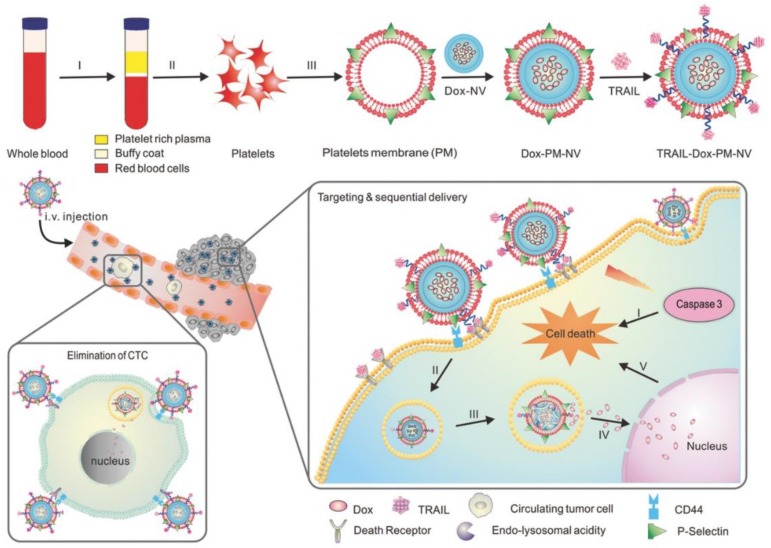
Schematic illustration of loading drugs (tumor necrosis factor (TNF)-related apoptosis inducing ligand, TRAIL and doxorubicin, Dox) into platelet membrane-coated nanoparticles for targeting and sequential drug delivery. Reprinted with permission from [62].

Platelets are tiny blood cells and key players in hemostasis and thrombosis, where they are naturally recruited to sites of vascular injury to trigger a cascade that leads to clot formation, helping the body form clots to stop bleeding [6]. In addition, emerging evidence have found that platelets are versatile cells that are also involved in many other pathophysiological processes, including innate and adaptive immune responses, atherosclerosis, angiogenesis, and tumor metastasis [7,59]. For example, recent studies have found that tumor cells detach from the primary site and intravasate into the blood vessel to form circulating tumor cells; platelets have the capability to recognize and interact with circulating tumor cells to form aggregates, which promotes tumor cell survival in circulation, thus leading to tumor cell metastasis [60,61]. Inspired by those above inherent abilities of platelets, platelet-based drug delivery strategies have been greatly explored in many types of disease settings [1,2]. Dr. Gu and his team engineered different platelet membranes and then coated them onto the surface of polymeric nanoparticles for the targeted delivery of anticancer drugs. In order to achieve a sequential and site-specific delivery of tumor necrosis factor (TNF)-related apoptosis inducing ligand (TRAIL) and doxorubicin (DOX) for an enhanced therapeutic efficacy [62], Hu et al. isolated platelet membrane and conjugated TRAIL onto the surface of platelet membranes via the bifunctional group sulfo-SMCC (Figure 3). The TRAIL-coupled platelet membrane was used to coat PLGA containing DOX to generate a dual payloads-loaded platelet membrane coated nanovesicle (TRAIL-Dox-PM-NV). The results showed that TRAIL-Dox-PM-NV achieved the site-specific delivery of an extracellularly active drug and an intracellular functional small-molecular drug, which effectively eliminated the circulating tumor cells in vivo and inhibited the development of tumor metastasis, thus leading to enhanced antitumor efficacy. Later on, Hu et al. engineered platelet membrane-coated nanovesicles for the enhanced treatment of multiple myeloma and thrombus [63], where fibrinolytic enzyme tPA was conjugated on the platelet membrane via biotin–streptavidin affinity that was further decorated by a targeting ligand alendronate through the sulfo-SMCC linker. The engineered platelet membrane was used to coat dextran nanoparticles containing the proteasome inhibitor bortezomib to achieve enhanced treatment efficacy.

In addition to engineered platelet membrane-coated nanovesicles, directly engineering whole platelets as delivery carriers has also been endeavored. Dr. Gu’s lab chemically conjugated anti-programmed-death ligand 1 (aPDL1) to the surface of resting platelets (or non-activated) via a bifunctional maleimide linker and found that the binding of aPDL1 to non-activated platelets was highly stable, while the release of aPDL1 could be significantly promoted upon platelet activation [64]. This enhanced release of aPDL1 possibly resulted from platelet-derived microparticles, which are derived from the plasma membrane of activated platelets and bind to the cancer cells. Animal studies demonstrated that the administration of platelet coupling aPDL1 substantially accumulated in the surgical site and significantly prolonged the overall survival.

Furthermore, by taking advantage of HSC’s homing capability to the bone marrow where locally released aPD-1 could enhance immune response, Dr. Gu’s lab conjugated aPD-1 coupling platelets to the surface of HSC through a click reaction [65]. To achieve that design, they treated HSC with N-azidoacetylgalactosamine-tetraacylated (Ac4GalNAz) to obtain Ac4GalNAz-modified HSC, and further reacted with functionalized aPD-1 coupling platelets, where the amine groups on the platelet surface were conjugated with dibenzocyclooctyne-PEG4-N-hydroxysuccinimidyl ester (DBCO-PEG4-NHS ester). The results showed that the engineered platelet–HCS assembly actively delivered aPD-1 to the bone marrow, increased the number of active T cells, and induced a potent immune response, thus effectively inhibiting leukemia growth while mitigating toxicities.

### 3.3. Cancer Cells

Cancer cells mostly derive from genetic changes and differ from normal cells in the body in many ways, including continuing to grow, immortality, and having the ability to metastasize to other regions of the body. The surface engineering of cancer cells has been leveraged for both therapeutic and diagnostic purposes. For cancer treatment, whole killed tumor cells provide a comprehensive source of tumor-associated antigens (TAAs) and have been utilized as the primary immunogens. However, in practice, whole killed tumor cell vaccines generally are poorly immunogenic, and they tend to fail to induce potent protective immunity. To overcome this limitation, the cell surface engineering of tumor cells with a variety of immune modifiers and costimulatory agents can enhance the immune recognition and responsiveness of the tumor cell vaccines. Shirota et al. covalently conjugated immunostimulatory oligodeoxynucleotides containing unmethylated cytosine–guanosine (CpG) dinucleotides (CpG–OND) to apoptotic tumor cells via an amine-to-amine cross-linker [66]. Results indicated that CpG–OND conjugation enhanced the uptake of TAAs by dendritic cells, induced costimulatory molecule expression, and promoted the production of antitumor cytokines. Vaccination with CpG–OND-conjugated tumor cells in an animal model triggered the potent immunity against multiple tumor cells, reduced the growth of established tumors, and prevented their metastatic spread. However, a limitation of this direct chemical linking of immunological adjuvants onto the surface of cancer cells is that the immunogenic function of some adjuvants might be compromised during the chemical conjugation process. To circumvent the potential problem of adjuvant inactivation, first packaging those immunogenic adjuvants into polymeric nanoparticles and then chemically linking these cargo-loaded nanoparticles to cancer cells could be a promising cancer vaccine platform. Dr. Salem’s group loaded Toll-like receptor-9 ligands into PLGA nanoparticles and then functionalized those nanoparticles onto the surface of irradiated tumor cells via streptavidin–biotin cross-linking [9]. The confocal microscopy results revealed that the majority of nanoparticles were surface bound to tumor cells as opposed to being internalized. They tested the cancer cell–nanoparticle hybrid in both prostate cancer and melanoma models. The cancer cell–nanoparticle hybrid had a significant therapeutic effect in a prostate tumor model, while it did not result in further improvements of the therapeutic effects in a melanoma model, which suggested that a need for the further optimization of the vaccination regime and/or adjuvant composition.

The early detection of cancer cells is vital for cancer diagnosis and targeted therapy. Engineering and converting some tumor-associated or -specific surface receptors into some detectable reporters provide a powerful tool to distinguish cancer cells [67]. Dr. Chen’s group used the high metabolic activity of cancer-overexpressed enzymes (histone deacetylase and cathepsin L-responsive acetylated azidomannosamine) to design an enzymatically activatable Ac4ManAz analog, which was subjected to a metabolic labeling process to form azido-containing sialic acid, and further conjugated this analog to glycoproteins highly expressed on the cancer cell surface [68]. Both *in vitro* and in vivo studies verified the efficient cancer cell labeling potency using DBCO-conjugated fluorescent dye. Furthermore, they successfully expanded the application of this cancer-selective labeling of azide groups for targeted cancer therapy using DBCO-conjugated doxorubicin.

### 3.4. Monocytes/Macrophages

Monocytes are an important type of leukocytes (or white blood cells) that represent 10% of the leukocytes in humans and play key roles in inflammation, pathogen challenge, and homeostasis. Monocytes stay circulating in the blood for a few days and migrate into various tissues where they differentiate into different resident macrophages. Since the conversion from monocytes to macrophages is a highly dynamic process in vivo, they share many common biological functions [69,70]. Hence, here we discuss the surface engineering of monocytes/macrophages together. The potential advantage of using monocytes/macrophages for drug delivery carriers is multifaceted [71,72]. For example, monocytes/macrophages can be actively recruited to the inflammatory tissues; monocytes/macrophages have the ability to cross complicated biological barriers, migrate, and penetrate into some hypoxic or poorly vascularized parts inside tumors; the long lifespan of monocytes/macrophages prolongs the circulation of encapsulated therapeutics or nanoparticles through the avoidance of immune recognition.

In order to enhance the antigen-capturing ability, recognition, and presentation of macrophages to tumor cells, Sugimoto et al. utilized the metabolically mediated indirect conjugation method to modify the surface of live macrophages with tumor-targeting nucleic acid aptamers. To achieve this, *N*-methacryloyl mannosamine was first fed to macrophages and then metabolically converted into the methacryloyl group that was included in the sialic acid on the cell surface. The methacryloyl group-modified macrophages were conjugated with thiol-terminated aptamers through a thiol–ene click reaction. The resulting engineered macrophages showed an increased capture of apoptotic tumor cells and presentation of major histocompatibility complexes from tumor cells, which were promising for anticancer immune activation [73]. In order to use macrophages’ inherent hypoxia-targeting ability and minimize the toxic effect of encapsulated anticancer drugs on the viability and hypoxia-targeting ability of the macrophage itself, Dr. Yang and his team engineered the surface of the macrophage with nanoparticles containing chemotherapeutics to generate a macrophage–nanoparticle hybrid vehicle for hypoxia-targeted drug delivery [74]. In their study, sialic acid residues on the RAW264.7 macrophage surface were modified with sodium periodate to generate aldehydes. Aldehydes reacted with the amine group of the PEGylated polyamidoamine (PAMAM) dendrimer to form Schiff bases-mediated transient macrophage–nanoparticle hybrid vehicles. Schiff bases can be further reduced to stable secondary amine linkages using sodium cyanoborohydride. Their results showed that the stable amine linkages retained nanoparticles on the macrophage surface for a longer time and had a larger amount of nanoparticle coating than the transit Schiff bases linkages did. This proof and concept study promises the microphage–nanoparticle hybrid vehicle as targeted drug carriers to hypoxic areas.

**Figure 4:**
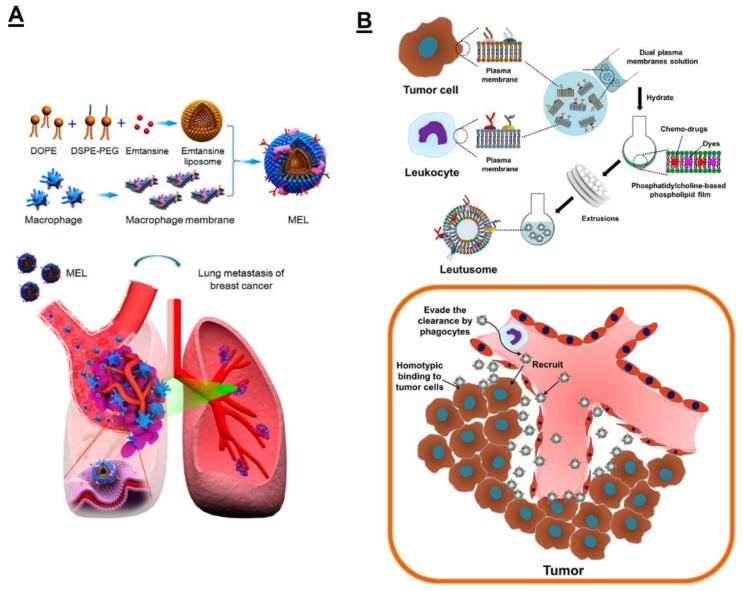
(**A**) Macrophage membrane-derived vesicle was modified with PEGylated liposome containing chemotherapeutics emtansine for enhanced tumor targeting property. Reprinted with permission from [75]. (**B**) The tumor cell membrane was integrated into the macrophage membrane to construct leutusome. The leutusome was found out to have prolonged circulation time, low non-specific phagocytic uptake by the reticuloendothelial system (RES), and strong tumor infiltration as well as preferential tumor cell uptake due to the dual cell membrane camouflage. Reprinted with permission from [78].

In addition to whole monocytes/macrophages as drug carriers, the monocyte/macrophage membranes were also engineered to be drug delivery vesicles. Due to the native homing property of macrophages to the tumor microenvironments, Cao et al. engineered the macrophage membrane by loading the anticancer drug emtansine to target the lung metastatic sites of breast cancer [75]. To achieve this, they fused the macrophage membrane with liposomal emtansine via the liposome fusion method (Figure 4A). This macrophage membrane-derived nanovesicle targeted emtansine to metastatic cells and enhanced the inhibitory effects on cell viability. The in vivo studies showed that macrophage membrane-derived nanovesicle encapsulating emtansine produced a notable inhibitory effect on the lung metastasis of breast cancer. Through the liposome fusion method, membranes from different cell types will be integrated into a new chimeric membrane, which inherits those multiple biofunctions from those parental cells [76,77]. Based on this concept, He et al. [78] engineered a macrophage membrane by incorporating it with a tumor cell membrane to endow the cancer cells’ homotypic targeting ability to engineered macrophage membrane-derived nanovesicles (termed leutusomes), which not only possessed the infiltrating property from macrophages, but also the active homotypic targeting property from cancer cells. To test that hypothesis, they loaded the anticancer drug paclitaxel into the leutusome (Figure 4B) and demonstrated that leutusome exhibited a prolonged circulation time and efficiently accumulated at the tumor site (79.1% ± 6.6% injected dose per gram of tumor). Furthermore, paclitaxel-encapsulating leutusomes potently inhibited tumor growth while not leading to systemic adverse effects as free paclitaxel induces.

### 3.5. Lymphocytes

Lymphocytes are one of the types of white blood cells and play pivotal functions in the immune system. Lymphocytes originate from stem cells in the bone marrow and primarily exist in the blood and lymphoid tissues. There are two major categories of lymphocytes, which are known as B lymphocytes (B cells) and T lymphocytes (T cells). After being produced in bone marrow, some lymphocytes will stay inside the bone marrow and develop into B cells. Meanwhile, some lymphocytes travel through the blood to the thymus and mature into T cells there. Others remain in the bone marrow, where they become B cells. These cells are responsible for the adaptive immune system and work together to defend the body against some threatening foreign substances, such as viruses, bacteria, and cancer cells. The function of B cells is to make antibodies, which are proteins produced by the immune system to fight antigens. The job of T cells is to play a critical role in cell-mediated immunity to help the body kill cancer cells. They patrol inside the body, screen out the cells that have been infected by viruses or become cancerous, and destroy them [11]. Thus, both T cells and B cells play important functions in immune response and are involved in numerous protective activities, including virus clearance, antigen detection, disease sites infiltration, and abnormal cell destruction. Collectively, those above-mentioned merits make lymphocytes promising candidates for drug delivery. Recently, adoptive T cell transfer (ACT) is a new area of transfusion medicine involving the infusion of lymphocytes to mediate antitumor, antiviral, or anti-inflammatory effects. However, the concurrent delivery of adjuvant drugs in the ACT, such as gamma chain (γ_c_) receptor cytokines or transforming growth factor-β signaling inhibitors, often needs to be maintained at high systemic levels for efficacy [79,80]. This would lead to dose-limiting toxicities for these cytokines due to their generally pleiotropic activities, which have limited their clinical use [81]. Some recent commercial approvals of chimeric antigen receptor (CAR)-T cell products to treat leukemia and lymphoma have greatly advanced this field from bench to bedside [30].

In comparison to the complexity of genetically engineering autologous T cells to express and produce special receptors on their surfaces for CAR-T therapy, the surface engineering of lymphocytes to trigger enhanced immune reaction could be a novel alternative approach for immuno-oncology.

In order to focus adjuvant drug action on the adoptive T cells to maximize donor cell efficacy and in vivo persistence, Dr. Irvine and his team developed an alternate strategy for adjuvant drug delivery in cell therapies [82]. They chemically conjugated submicron-sized cytokines-loaded synthetic nanoparticles onto the plasma membrane of donor cells, enabling continuous pseudoautocrine stimulation of their cellular carriers in vivo. To achieve this, cytokines-carrier synthetic nanoparticles (a backpack strategy) were chemically conjugated to the surface of therapeutic cells via maleimide–thiol coupling, followed by in situ conjugation to thiol-terminated polyethylene glycol to quench the residual reactive groups of the nanoparticles. The results showed that coupling of up to approximately 170–200 nm lipid nanoparticles to the cell surface was nontoxic and did not alter key cellular functions, including killing target cells, proliferating and secreting cytokines. *In vivo* studies demonstrated that nanoparticle-decorated T cells efficiently targeted surface-tethered nanoparticles into antigen-expressing tumors, markedly amplified the proliferation of their cellular carriers, and significantly prevented tumor growth with all mice alive 30 d after T cell treatment. A recent study from Dr. Irvine’s lab aimed to improve the efficient release of cytokines from the nanoparticle backpacks, allowing for an enhanced autocrine stimulation of transferred T cells [83]. They synthesized a reduction-responsive degradable cross-linker (a disulfide-containing bis-N-hydroxy succinimide cross-linker) to construct protein-based nanoparticles using two clinical therapeutic candidates, such as a human IL-2Fc (a fusion version of IL-2 and an antibody Fc fragment) and a human IL-15 super-agonist complex. They conjugated those redox-responsive nanoparticles to T cells. When the T cells get activation in vivo, this disulfide cross-linker among the nanoparticles will be cleaved, thus leading to the rapid release of intact proteins cargo via a self-immolative reaction and thus enhancing T cell persistence and function. In addition, both stable cell surface retention and the release of cytokines from nanoparticles are important for maximal stimulation to adoptive cells. Nanoparticle backpacks should stay onto the cell surface and should not be internalized by the carrier cells [82]. In order to stabilize those backpacks onto the T cells, they screened out a series of monoclonal antibodies as specific cell anchors for backpacks and found out that anti-CD45 modification to backpacks exhibited prolonged cell surface retention, compared with other antibody-modified or non-modified backpacks. The proof-of-concept study showed that the backpacked T cells maintained an efficient stimulation of T cells for at least a week in culture condition, preferentially expanded T cells 16-fold in tumors, and allowed at least an eightfold higher doses of cytokine to be systemically administered without detectable toxicities [83].

**Figure 5:**
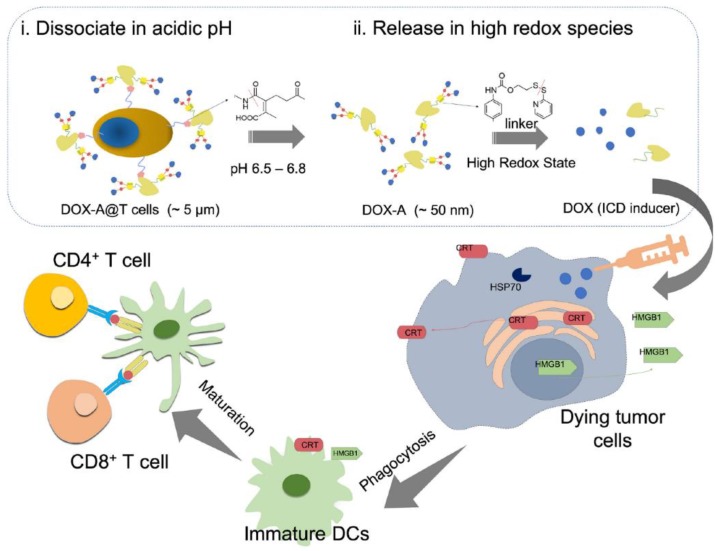
Schematic design of nanoengineered lymphocytes as the delivery vehicle via the self-immolative mode to increase tumor apoptosis and the immunogenic cell death of doxorubicin, thus efficiently potentiating immune response and suppressing the tumor growth. Reprinted with permission from [85].

In addition to the application of lymphocytes surface engineering in adoptive cell therapy, it also has been endeavored for active targeting drug delivery carriers. Given the fact that healthy lymphocytes can be programmed to phenocopy the dissemination of the tumor cells into compartments, which are poorly accessible for systemic chemotherapy, Huang et al. used lymphocytes as “Trojan horses” to deliver SN-38-loaded lipid nanoparticles via a maleimide–thiol reaction [84]. The cell studies showed that this loading method mediated the efficient killing of lymphoma cells without causing acute toxicity to the carrier cell. *In vivo* data demonstrated that the nanoparticle-decorated T cells actively deliver SN-38 to lymphoid organs and showed the most marked tumor growth suppression, exhibiting a significant reduction (60-fold smaller) in tumor burden relative to the free drug or untreated animals. Liu et al. devised a novel pH-sensitive bond to chemically modify the self-immolative chemotherapeutics–albumin complex onto the surface of T lymphocytes for enhanced tumor penetration and improved antitumor efficacy [85]. In their design (Figure 5), the engineered T lymphocytes can act as a shuttle to efficiently deliver doxorubicin–albumin conjugates to peripheral cells of the tumor but not the tumor core region. The doxorubicin–albumin complex was first cleaved under the more acidic tumor microenvironment (pH 6.5). Later on, the doxorubicin–albumin complex penetrated to the tumor core region and was ultra-smaller compared to the micro-size of engineered T lymphocytes. Once being internalized by tumor cells, the doxorubicin–albumin conjugate underwent a self-immolative reaction to release doxorubicin under the action of an abundant intracellular redox species. This high cellular concentration of doxorubicin exerted potent therapeutic benefits via both concentration-dependent chemotherapy and immunogenic cell death without systemic toxicity.

### 3.6. Stem Cells

Stem cells are special cells and can differentiate into many different cell types that make up the body. In some cases, they can replace cells that are damaged or lost. Stem cells originate from two main sources: embryonic stem cells and adult stem cells. These embryonic stem cells are pluripotent, suggesting that they can turn into more cell types than adult stem cells could. The embryonic stem cells used in today’s biomedical researches are unused embryos that come from an *in vitro* fertilization procedure and are donated to science. The use of embryonic stem cells is still highly controversial. In August 2001, President Bush prohibited scientists in the United States from developing new embryonic stem cell lines, instead only allowing scientists to work with embryonic stem cell lines already in existence. In March 2009, President Obama issued an executive order to remove some restrictions on embryonic stem cells. Nevertheless, the research for embryonic stem cells in United States heavily lagged behind the progress in many other countries [86]. Generally, adult stem cells can be grouped into two types. One type of adult stem cells derive from fully developed tissues, such as the brain, skin, and bone marrow. There are only tiny numbers of this type of adult stem cells in these tissues, and they are more likely to develop into a specialized type of cells. The second type is induced pluripotent stem cells that have been manipulated in a laboratory to take on the pluripotent characteristics of embryonic stem cells. Several types of adult stem cells have been studied for various biomedical applications, including mesenchymal stem cells (MSC), neural stem cells (NSC), and hematopoietic stem cells (HSC). What we discuss here mainly focuses on the application of MSC. The research of MSC is attractive because they can differentiate into various types of cells, including adipocytes, osteoblasts, and chondroblasts. In addition, MSC can promote angiogenesis and have immunomodulatory effects [14]. Currently, over 100 clinical trials with MSC are being tested to regenerate damaged tissues and treat inflammation [13].

**Figure 6:**
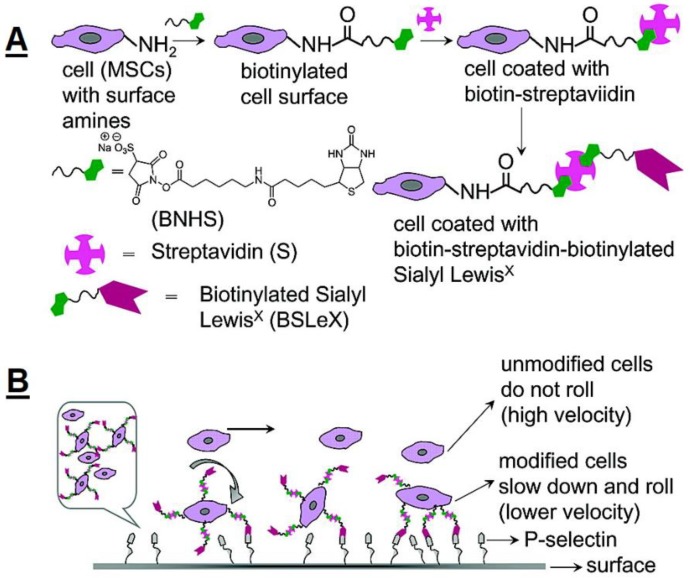
(**A**) Modification of mesenchymal stem cells (MSC) by sialyl Lewisx (SLeX) mediated by biotin−streptavidin conjugation. (**B**) Schematic illustration for the rolling of SLeX modified MSC on a P-selectin coated surface. Reprinted with permission from [92].

Stem cells have been demonstrated to possess inherent tumoritropic migratory property, and this has been utilized in cancer therapies. Dr. Anderson’s lab designed the conjugation of neutravidin-modified nanoparticles with both biotinylated human bone marrow-derived MSC [87]. The human MSC attached with nanoparticles did not lose their capacity to sense and preferentially target tumor spheroids grown in 3D collagen gels. The enhanced engraftment of MSC within ischemia myocardial tissue has been demonstrated to prevent the formation of fibrous scars and enhance tissue regeneration. However, a significant limitation is the inability of administrated MSC to reach target tissues with high efficiency. The native cell–cell interactions under dynamic flow conditions in vivo have significantly directed the design of exogenous therapeutic cells that target inflamed or injured tissues. Dr. Dennis’s lab modified MSC to specifically target endothelial cells via binding to intercellular cell adhesion molecule-1 (ICAM-1) [88]. In their studies, they coated MSC with palmitated protein G (PPG) followed by antibodies to ICAM-1, which showed a 40-fold increase in cell binding on ICAM-I coated coverslips over PPG-only controls. Dr. Karp’s team demonstrated that nucleic acid aptamers, when chemically attached onto the MSC surface, served as artificial “adhesion ligands” and enabled them to bind under dynamic flow conditions to endothelial cells or leukocytes, thus repairing injured tissues [89]. Other several examples of engineering MSC to deliver a large quantity of viable cells to a tissue of interest also came from Dr. Karp’s group [15,90,91,92]. They functionalized the surface of MSC with a nanometer-scale polymer construct containing sialyl Lewisx (sLex), which is the active site of P-selectin glycoprotein ligand 1 that is found on the surface of leukocytes and mediates cell rolling within inflamed tissue (Figure 6). The sLex-modified MSCs exhibited a robust rolling response on an inflamed endothelium in vivo and actively homed to inflamed tissues with higher efficiency compared with unmodified MSC.

In the other inflammation-related diseases such as inflammatory bowel disease, clinical studies have shown that MSC have the potential to resolve inflammation owing to their immunosuppressive capabilities [27,93]. Dr. Dennis’s lab aimed to coat MSC with antibodies against addressins to enhance their delivery to the sites of inflammation in colon and thereby improve therapeutic outcomes [94]. Another application of MSC is to treat congenital and acquired bone diseases. However, their effectiveness is highly limited by the poor bone-targeting property of administrated MSC. Given that hematopoietic stem cells express E-selectin ligands that are crucial for homing to bone [95], Dr. Sackstein et al. enzymatically processed MSC using an α-1,3-fucosyltransferase preparation to convert native CD44 glycoprotein on MSC into hematopoietic cell E-selectin/L-selectin ligand, which conferred potent tropism to bones without side effects on cell viability or multipotency [96].

In addition to the treatment of surfaced engineered MSC, it was also used as a signaling sensor. Dr. Karp et al. conjugated a fluorescent sensor to the MSC membrane to detect signaling molecules in the cellular environment. When bound to a signaling molecule platelet-derived growth factor (PDGF), the changed conformation of the sensor made the dyes attach closer to each other and led to a signal. This sensor can quantitatively detect PDGF with high spatial and temporal resolution [97].

### 3.7. Bacteria

Bacteria are ubiquitous microorganisms that lack a membrane-bound nucleus and other internal structures. Bacteria display exceedingly diverse metabolic capabilities and can use almost any organic compound. Most bacteria are harmless and are beneficial ecological agents, while some bacteria can cause diseases by evading immune responses in humans, animals, or plants. Bacteria have developed unique strategies to evade the host’s immune system and enter a target cell, which enable bacteria to be a promising drug delivery carrier that may be able to overcome some of the limitations of conventional drug delivery carriers.

It has been discovered that some strains of bacteria, including *Salmonella typhimurium*, *Clostridium beijerinckii*, and *Bifidobacterium bifidum*, have a natural tumor-targeting ability and they specifically colonize hypoxic areas of tumors that cannot be achieved by most chemotherapeutic drugs. To use the tumor-targeting feature, some bacteria have been genetically engineered to synthesize and secrete some therapeutically active substances such as cytokines, enzymes, and antibodies, which alone can act as potent antitumor agents. Another novel approach is the development of microbots that are bacteria conjugated with nanoparticles containing antitumor agents on their surface. The microbots successfully enter tumor cells and release nanoparticles, resulting in intracellular drug delivery [98,99,100,101,102]. For example, Dr. Behkam et al. used a weakened version of salmonella (VNP20009) as tumor-targeting bacteria and conjugated PLGA nanoparticle to the surface of modified salmonella via streptavidin–biotin noncovalent affinity-based bonds [16]. The results showed that this engineered bacteria coupled with nanoparticles had a similar tumor infiltration property to the native bacteria. This microbots design has also been demonstrated as effective to deliver DNA-based model drug molecules. Dr. Bashir et al. conjugated nanoparticles that were loaded with plasmid DNAs to the surface of the bacteria via a biotin–streptavidin interaction [17]. The microbots successfully delivered their cargos of nucleic acid into target cells, which resulted in the subsequent transcription and translation of the target proteins. Moreover, Cao et al. used the erythrocyte membrane to coat bacteria to reduce some common side effects associated with live bacterial drug delivery carriers, including high inflammatory response, rapid elimination by macrophages within RES, and low accumulation in target tissues [103].

In addition to live bacteria as drug delivery systems, bacteria ghosts as nonliving bacteria have been made for therapeutic delivery functions. In bacterial ghosts, cytoplasmic components including genetic materials are removed from Gram-negative cells, while preserving the morphology and surface antigenic structures [104]. Despite the depletion of cytoplasmic contents, the intrinsic surface components on bacteria ghosts such as flagella, fimbriae, and polysaccharides allow for the conjugation or tethering of a range of payloads and enable an intrinsic ability to target various cells such as dendritic cells [105], macrophages [106], and cancer cells [107]. In addition to their antigen-carrying feature, bacterial ghosts also retain some intrinsic immune adjuvant properties that are derived from bacteria membrane components such as peptidoglycan and lipopolysaccharides, which make bacteria ghosts excellent vaccine systems [108]. Drugs such as chemotherapeutics and adjuvants can also be loaded to the internal lumen or periplasmic space of the carrier [109].

**Figure 7:**
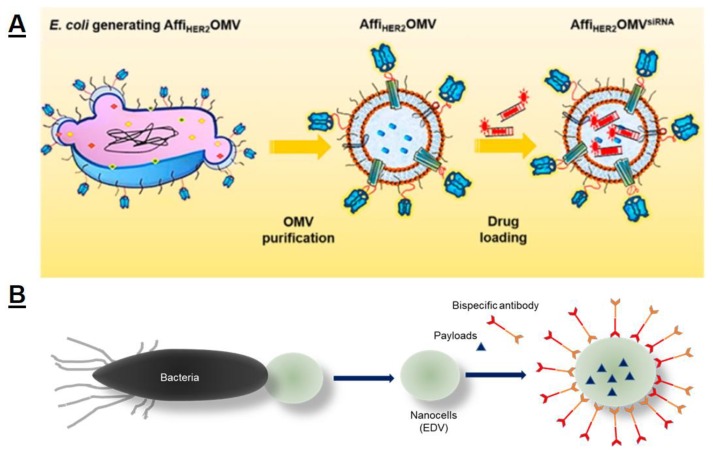
(**A**) Schematic representation of outer membrane vesicles (OMVs) expressing human epidermal growth factor receptor 2 (HER2)-specific affibody, and further loaded with TAMRA-labeled siRNA. Reprinted with permission from [111]. (**B**) Schematic illustration of the EnGeneIC Dream Vector (EDV) platform developed by EnGeneIC Ltd. EDV is a type of nanoparticle (around 400 nm) formed by a genetically modified bacterium during its division. A single EDV can be packed with a larger amount of various payloads, including anticancer drugs and nucleic acid-based therapeutics. The surface of EDV can be further modified with a bispecific antibodies, in which one arm recognizes the O-antigen component on the EDV surface lipopolysaccharides (LPS) molecule and the other arm is a cell-surface receptor specific for the tumor cells to be targeted. TAMRA: tetra methyl-rhodamine, siRNA: small interfering RNA.

Bacterial outer membrane vesicles (OMV) are vesicles of lipids released from the outer membranes of Gram-negative bacteria. OMV share a great similarity in biochemical profiles with their parent cells and have been utilized for drug delivery [110]. Deriving from bioengineered Escherichia coli strain, Gujrati et al. modified human epidermal growth factor receptor 2 (HER2)-specific affibody onto the membrane of OMV as a tumor-targeting ligand. Later, the HER2 expressing OMV were loaded with the therapeutic siRNA targeting kinesin spindle protein (Figure 7A). The systemic injection of HER2-modified OMV containing siRNA caused targeted gene silencing and induced highly significant tumor growth regression in an animal model [111].

EnGeneIC Dream Vector (EDV) is a bacterially derived nanocell platform that was developed by EnGeneIC Ltd. EDV is formed during a genetically modified bacterium divides at its pole as well as centrally. This nanocell does not contain a chromosome and has the average size of 400 nm. EDV has been demonstrated as a powerful delivery platform for drug, siRNA, or microRNA. In addition, the targeting of EDV was further achieved using bispecific antibodies, in which one arm recognizes the O-antigen component on the EDV surface LPS, and the other arm is a cell-surface receptor specific for the tumor cells to be targeted—for example, the human epidermal growth factor receptor 2 (HER2) or the epidermal growth factor receptor (EGFR) on ovarian and breast cancer cells, respectively (Figure 7B). Those preclinical studies demonstrated that targeted EDV directly targeted chemotherapeutics to tumor cells and effectively killed tumor cells with minimal toxicity, while simultaneously stimulating the immune system’s innate and adaptive antitumor response. Among these, the EGFR–targeted, doxorubicin-loaded EDV nanocell is currently being tested in a phase 1 trial (NCT02766699) [112,113].

Although bacteria-based drug therapies have been shown to achieve successful results in vivo, they are not currently used as a standard route of drug administration due to safety concerns. Further thorough investigations, such as the potential immunogenicity of attenuated bacteria and their in vivo fate of the bacteria, are needed before they are completely accepted for clinical applications.

### 3.8. Extracellular Vesicles

Extracellular vesicles (EVs) are lipid bilayer-based nanoparticles within the size range of 30–1000 nm that are naturally secreted from cells into the extracellular space. Unlike cells, EVs cannot replicate. The main biological functions of EVs are to shuttle a variety of cargoes, such as proteins, nucleic acids, lipids, metabolites, and even organelles from parent cells to the others, and exchange genetic information between cells [22]. The shuttling cargoes and carrying messages of EVs are dependent on the type and state of the parent cells [114]. Based on the different parent cells, the formation mechanism and various sizes and diverse EVs subtypes have been proposed, such as exosomes, microvesicles, apoptotic bodies, and others. Exosomes are produced in the endosomal compartment of most eukaryotic cells, where the multivesicular body fuses with the plasma membrane and buds directly from the plasma membrane. Whereas microvesicles and apoptotic bodies are derived from plasma membrane shedding and cells undergoing apoptosis, respectively [115]. In addition to the importance of EVs in cell-to-cell communications, some other pathophysiological roles of EVs are currently well recognized in multiple disease models, including cancer, cardiovascular, liver, infectious, and neurodegenerative diseases. The EVs’ excellent merits, such as their distinctive structure feature, endogenous origin, and native targeting property, have encouraged researchers to develop EVs-based therapeutic carriers by loading EVs with a wide spectrum of drugs, including anti-inflammatory [116], anticancer [117], and nucleic acid drugs [118,119,120,121].

Although EVs have emerged as novel therapeutic effectors in immune therapy, regenerative medicine, and drug delivery, recent studies found out that the biodistribution of injected unmodified EV revealed a rapid accumulation in organs of the RES such as the liver and spleen, and led to a lower target accumulation after systemic administration [122]. Thus, the targeting property of EVs can be further improved via some modifications, such as surface modifications [21,123].

There are generally two approaches to modify the surface of EVs with desired targeting properties. One can be produced from parental cells genetically engineered to express membrane-bound targeting ligands; the other one is generated by non-genetically modifying the surface of EVs with some targeting ligands through covalent conjugation or non-covalent conjugation. There are some are still big concerns with genetic engineering, such as its toxicity, potential mutagenesis, and ease of operation. By contrast, the relatively simple procedure has made non-genetic modification become more and more popular for EVs surface modification.

**Figure 8:**
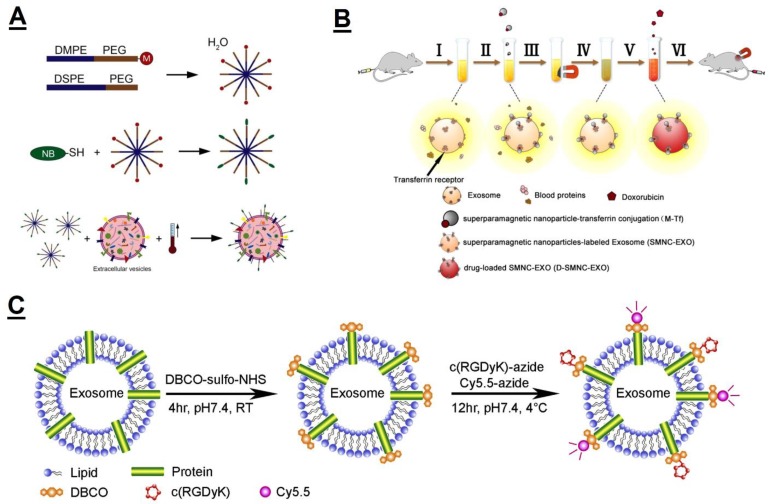
(**A**) Schematic illustration of isolated extracellular vesicles are modified with nanobodies via PEG-micellar post-insertion. Reprinted with permission from [124]. (**B**) Schematic representation of the construction and delivery of magnetic drug-loaded extracellular vesicles). Reprinted with permission from [126]. (**C**) Schematic depiction of conjugating c(RGDyK) and Cy5.5 fluorophore to exosomal amine groups by a click reaction. Reprinted with permission from [115]. c(RGDyK): the cyclo(Arg-Gly-Asp-D-Tyr-Lys) peptide.

In order to improve the tumor-targeting drug delivery of EVs, Dr. Schiffelers et al. utilized hydrophobic insertion to decorate the surface of EVs with targeting ligand EGFR, which was conjugated to phospholipid–PEG derivatives (EGFR-PEG-PL) [124]. To achieve this, a micelle was first constructed with EGFR-PEG-PL and mixed with EVs to form the targeting ligand-modified EVs (Figure 8A). The results showed that the modified EVs improved specific tumor cell binding *in vitro* and prolonged circulation time in vivo; however, the significant increment of modified EVs in tumors was not observed, which was possibly due to it being below the detection limit. Whether these PEGylated and targeted EVs can promote functional cargo delivery to targets through their unique composition remains to be elucidated in the future.

Through electrostatic interaction and hydrophobic insertion, Nakase et al. fused EVs with some commercially available cationic lipids and a pH-sensitive fusogenic peptide to enhance cellular uptake and the endosomal escape of EVs-loaded drugs [125]. Qi et al. used the non-covalent modification method to develop magnetic drug-loaded EVs (Figure 8B), which showed an enhanced tumor targeting under an external magnetic field and greatly suppressed tumor growth. To achieve that, they first coupled superparamagnetic magnetite colloidal nanocrystal clusters onto the surface of reticulocytes-derived EVs through transferrin–transferrin receptor interaction. Next, they loaded the magnetic EVs with DOX through a hydrophobic effect to form magnetic DOX-loaded EVs [126].

A novel method for the conjugation of ligands to the surface of EVs was developed via click chemistry. Smyth et al. conjugated alkyne-containing 4-pentynoic acid onto EVs membrane amines using the carbodiimide coupling. Next, the reactive alkyne base subjected to a second click chemistry reaction with an azide-tagged fluorophore. This conjugation did not affect the size of exosomes, nor did it alter the extent of exosome adherence/internalization with recipient cells [127]. In addition to the tumor targeting, the other targeting properties of EVs, such as the lesion region of the ischemic brain, were also explored. The c(RGDyK) peptide has been conjugated to the exosome surface by Tian et al. via a bio-orthogonal chemistry (Figure 8C). To conjugate EVs with c(RGDyK), they first introduced DBCO groups to the isolated EVs surface by cross-linking DBCO-sulfo-NHS. The NHS groups were reacted with the amino groups on the EVs’ surface to form covalent bonds. Second, the DBCO groups on the EVs reacted with azide-functionalized c(RGDyK) to form stable triazole linkages via copper-free click chemistry. The results showed that the targeted EVs dramatically accumulated in the lesion region and exhibited a significantly stronger anti-inflammatory effect when loaded with curcumin in the disease mouse model [115].

In order to simplify the labor-intensive procedures for current non-genetic EVs modifications, Wan et al. reported a novel method for targeting EVs. They covalently conjugated a nucleolin-targeting aptamer AS1411 to cholesterol-PEG followed by anchoring the compounds onto a living mouse dendritic cell membrane. Then, the modified cells were extruded by passing through microconstrictions to obtain EVs, which was subsequently loaded with paclitaxel under sonication. Both *in vitro* and in vivo results demonstrated that paclitaxel-loaded AS1411-EVs had the higher antitumor efficacy via enhanced accumulation in tumors [128].

## 4. Perspective and Conclusions

In this review, we briefly introduced major types of modification used for cell surface engineering (mainly non-genetic surface engineering) and then comprehensively focused on the recent progresses surrounding surface engineering in different cells and cell-based therapeutics. We highlighted the various non-genetic surface engineering strategies, such as covalent chemical conjugations and noncovalent physical conjugations, which can be utilized for modifying the surface of cells or cell-based therapeutics to render them with new functionalities, including the targeting properties, therapeutic activities, and enhanced pharmaceutical profiles. We specifically focused on several intensively investigated cell types, including erythrocytes, platelets, cancer cells, leukocytes, stem cells, and bacteria. Despite the promising data in research and early preclinical studies, the non-genetic surface engineering of cells or cell-based therapeutics is still in its infancy and faces some unmet challenges that restrict their further translation into clinic. This extremely low translational chance in this field can be partly described by our previous review surveying the clinical translation of cancer nanomedicines, since cells or cell-based therapeutics and some elements involved in this field share many common features with nanomedicines [129]. Whereas, there are some other challenges specific to this field, based on the distinctive features of cells or cell-based therapeutics as discussed above. One important challenge is how to reach the long-term stability of surface modification. As live cells maintain homeostasis by renewing their membranes, the introduced or modified molecules through non-genetic modification might be removed by the shedding or internalization of the renewing plasma membrane. To address that concern, some novel cell-based therapeutic modalities, such as cell membrane-derived therapeutics and extracellular vesicles, could provide some alternative options. The second limitation is that the potential effects of cell surface-modified materials and modification techniques on some crucial cellular functions, including intravascular transport, cell–cell interactions, and phenotypic activities, need a systematical study. Last but not the least is the potential cellular immune activation of surface-modified materials, which will convert the modified cells into antigens and then lead to the early elimination of injected cells from the body.

Despite those challenges, continuous technological innovations and multidisciplinary collaborations provide opportunities for the further advancement of non-genetic cell surface engineering as a clinical tool to endow cells with enhanced therapeutic efficacy. In order to eliminate the laborious cell expansion processes and negative impacts of non-genetic modification methods on the live cellular function, in vivo cell engineering methodologies should be highly desirable to develop. In addition, the development of novel biocompatible materials, such as functional nucleic acids and targeting aptamers, hold the promise to accelerate the implementation of cell surface engineering in clinic. Moreover, the non-genetic surface engineering of different cells or cell-based therapeutics should be personalized and investigated individually, since the different cells possess different biological functions and different responses to surface engineering. Furthermore, the development of some novel conjugation strategies that have the ability to conveniently conjugate functional materials or drugs to cells (such as copper-free click reaction-directed) and/or to efficiently release the drugs triggered by some external stimulus (light or temperature) and/or internal stimulus (enzymes, pH, and redox condition) will hold promise for the future of cell membrane engineering. Although most of the non-genetic surface engineering strategies described in this review are still in their infancy and are mainly restricted to preclinical studies, there is one novel form of cell-based therapeutics (bacteria-derived) that is being tested in clinical trials. EnGeneIC’s lead candidate EGFR-EDV-Dox, which is DOX-loaded bacteria-derived nanoshell with surface EGFR targeting modification, reported acceptable safety profiles and early efficacy evidence in a phase 1 trial. Currently, its antitumor efficacy is being extensively investigated in a phase 1 trial. We believe that the commercial success of cells or cell-based therapeutics modified with non-genetic cell surface engineering will come true in the near future. In summary, non-genetic surface engineering technologies have shown an innovative potential to reduce the toxicity issue associated with genetic engineered cell-based therapeutics (such as CAR-T) and enhance their therapeutic efficacy, which have shown a broad spectrum of biomedical applications, such as genetic engineering, drug delivery, and regenerative medicine as well as bioimaging.
